# Internet use and its association with physical activity among adolescents in Beni Municipality, Myagdi, Nepal

**DOI:** 10.1371/journal.pone.0302456

**Published:** 2024-05-03

**Authors:** Shristi K. C., Hari Prasad Kaphle, Nirmala Neupane, Samjhana Baral

**Affiliations:** School of Health and Allied Sciences, Pokhara University, Pokhara, Nepal; National Cheng Kung University College of Medicine, TAIWAN

## Abstract

**Introduction:**

Adolescence is a critical phase marked by distinct health and developmental needs and rights. It represents a pivotal time for the acquisition of knowledge, skills, emotional regulations, and relationship management. However, a recent surge in internet usage among adolescents has been observed, leading to a concerning decline in physical activity. This study aims to evaluate the level of physical activity and its correlation with internet use among adolescents.

**Materials and methods:**

A cross-sectional study, conducted within educational institutions in Beni, Nepal, utilized a self-structured questionnaire to collect data on physical activity levels and associated factors. The relationship between physical activity and independent variables was assessed through the chi-square test, and regression analysis was employed to quantify the strength of this association.

**Results:**

The study revealed that 75.3% of adolescents (out of 385) exhibited inadequate physical activity levels. Notably, late adolescents were more susceptible to insufficient physical activity than their middle adolescent counterparts were. Adolescents from nuclear families (UOR = 2.689, C.I = 4.074–22.822), those with higher monthly family income (UOR = 3.318, C.I = 1.209–9.105), and individuals using Wi-Fi (UOR = 1.801, C.I = 1.117–2.904) demonstrated a higher likelihood of internet addiction. Moreover, these groups were more prone to engage in inadequate physical activity (UOR = 1.1740, C.I = 0.938–3.226) compared to their counterparts not addicted to the internet.

**Conclusion:**

The study concludes that over two-thirds of adolescents were addicted to the Internet, and three-fourths were inadequately physically active, with late adolescents being more affected than middle adolescents. Inadequate physical activity was associated with various factors, including family type, monthly family income, monthly pocket money, father’s occupation, type of school, type of internet access, and Internet Addiction Test (IAT) score. Internet-addicted adolescents were found to be more likely to be physically inactive.

## Introduction

The World Health Organization (WHO) defines adolescence as the period spanning ages 10 to 19 [1], representing a transitional phase between childhood and adulthood marked by distinct health and developmental needs. This critical period offers a unique opportunity for the cultivation of knowledge, skills, and emotional intelligence. Adolescence is characterized by various changes, encompassing biological, physical, and neurodevelopmental transformations that significantly impact behavior and overall well-being [2].

WHO defines physical activity as any bodily movement produced by skeletal muscles that requires energy expenditure–including activities undertaken while working, playing, carrying out household chores, traveling, and engaging in recreational pursuits. The term "physical activity" should not be confused with "exercise", which is a subcategory of physical activity that is planned, structured, repetitive, and aims to improve or maintain one or more components of physical fitness [3]. Beyond exercise, any other physical activity performed during leisure time or as a part of a person’s work has a health benefit. Further, both moderate- and vigorous-intensity physical activity improve health. Globally, in 2010, 81% of adolescents aged 11–17 were insufficiently physically active. Adolescent girls were less active than boys, with 84% vs. 78% not meeting WHO recommendations [4].

The Internet, a global network of interconnected computer systems, plays a pivotal role in the lives of adolescents, serving as a conduit for education, communication, and personal growth. Internet use has been increasing in all age groups worldwide. Despite its global surge, especially among adolescents, internet usage brings potential risks, including sedentary behavior and diminished physical activity [5]. Especially among adolescents, the internet has become an important social context for development, but it also leads to risky behavior [6]. Even younger people who are financially unstable use the internet and mobile devices [7]. Engaging in excessive internet use can contribute to a sedentary lifestyle, reducing opportunities for physical activity. Internet use is associated with physical activity as high media use occurs with low physical activity behavior [8].

Although internet use has multiple positive impacts, there are opposing sides, such as access to all kinds of information, including self-distraction, risk-taking behaviors, and loneliness [9]. As one kind of specific internet use, problematic social media use had positive mediating effects together with fear of Corona Virus Disease 2019 (COVID-19) in the association between care burden and motivation of vaccine acceptance [10]. Young adults with internet gaming disorder have distinct problematic thoughts about gaming such as over-reliance on gaming to meet self-esteem needs, game rewards, and gaming as a method of gaining social acceptance highlighted the importance of addressing these cognitions in therapeutic interventions for the disorder [11].

In Southeast Asia, the prevalence rate of internet addiction and gaming disorders were 20% and 10.1% respectively [12]. Even during COVID-19, internet use led to various types of addiction including gaming addiction, social media addiction, food addiction, sex addiction, and shopping addiction [13]. Furthermore, problematic internet use is related to significant issues including fear of missing out, nomophobia, cyberchondria, cyberbullying, and health conditions such as attention-deficit hyperactivity disorder, schizophrenia [14], sleep problems [15], psychological distress, and quality of life [16].

Internet addiction among adolescents in Nepal has not been studied much so far, but a higher prevalence can be predicted as many adolescents are engaged in internet use [17]. While internet addiction among adolescents in Nepal remains relatively unexplored, the escalating prevalence of internet use among this demographic underscores the urgency for investigation. This study aims to assess the levels of physical activity, internet use, and interconnectedness among adolescents in Beni Municipality, Nepal.

## Materials and methods

### Study design and setting

This study employed an institutional-based cross-sectional analytical design in Beni Municipality; headquarters of the Myagdi district in Gandaki Province, Nepal. In Nepal, adolescents aged 15 to 19 comprise 10.17% (29,66,404) of the total country’s population. And Myagdi district alone has 9947 adolescents aged 15 to 19, according to the National Population and Housing Census (NPHC), 2021. Nestled in the hilly region, Beni Municipality provided a unique setting for exploring the physical activity of adolescents. Data collection spanned from May 2021 to June 2021.

### Study population and sampling

The study targeted secondary-level students aged 15–19 years from selected schools in Beni Municipality, Myagdi, Nepal. Utilizing a prevalence of internet use among Indian adolescents (35.6%) [18], with a 5% error and 95% confidence interval, a sample size of 350 was initially determined. Accounting for a 10% non-response rate, the final sample size was set at 385. Out of the 11 secondary-level schools in Beni municipality, four were randomly chosen using a random number app. Subsequently, stratified random sampling treated each section of the selected institutions as a stratum. The number of samples required from each stratum was determined proportionately. The final individual respondents for interviews were randomly selected using the lottery method.

### Study tool and data collection

A pretested self-administered questionnaire was employed to collect information in line with the study objectives. The socio-demographic section covered factors such as age, gender, educational status, and perceived socio-economic level. For the assessment of self-reported physical activity, the International Physical Activity Questionnaire (IPAQ) has been employed. IPAQ is an instrument designed to assess levels of physical activity, and short and long forms of the questionnaire have been developed based on self-report population surveys. The short form of the IPAQ was utilized to determine the physical activity levels of the individuals. The tool is recognized for its good validity and reliability [19]. The used version of IPAQ has been proven to have validity and reliability with co-relation factors of 0.77 and 0.95 [20, 21]. The tool has been reported to have Alpha Cronbach 0.7 and reliability was assessed by test re-test [22]. The criterion was that each activity should be done for at least 10 minutes. A score of "Metabolic Equivalent (MET) minute/week" is obtained by multiplying the minutes, days, and MET values (times of resting oxygen consumption). An individual can be categorized as physically inactive (<600 MET-min/week), low physical activity (600–3000 MET-min/week), and adequate physical activity (> 3000 MET-min/week) [23]. Physically inactive and low physical activity were collectively considered inadequate physical activity. In calculating energy consumption for physical activities, each activity’s weekly duration (minutes) and the MET energy values for the IPAQ were multiplied. Thus, for each individual, energy expenditures related to vigorous (8.0 MET/minute), moderate (4.0 MET/minute), walking (3.5 MET/minute), sitting (1 MET/minute), and total physical activities were obtained in MET-min /Week. IPAQ score was calculated by converting the activities that lasted more than 10 minutes to minutes before calculating MET minutes.

For example, if someone reports each of the walking, moderate and vigorous physical activities for 30 minutes 5 days a week. Then MET levels for walking will be (3.3*30*5) 495 MET-min/week, Moderate Intensity (4.0*30*5) 600 MET-min/week, Vigorous Intensity (8.0*30*5), 1,200 MET-min/week and total MET level will be (495+600+1200) 2,295 MET-min/week [24]. Total MET-min/week = (Walk METs*min*days) + (Moderate METs*min*days) + Vigorous METs*min*days)

One (1) MET is the rate at which you burn energy when sitting quietly. One MET is roughly equivalent to one kcal/kg/hour. Moderate-intensity activities fall in the range of 3–5.9 METs. Vigorous-intensity activities are 6.0 METs or greater.

The Internet Addiction Test (IAT), developed by Dr. Kimberley Young, consisting of 20 questions, was employed to assess internet addiction [25, 26]. The IAT has been widely translated and validated by various countries and has proven to consist of good internal validation values [27, 28]. The internal consistency of IAT in certain study populations was found to be excellent (Cronbach’s α = 0.834) [29]. The Cronbach’s alpha coefficient of the Internet Addiction Test in this study was 0.876. Scores on the IAT range from 0 to 100, which are assigned to four categories; a) 0–19 points for no addiction, b) 20–49 points reflecting mild internet use (one who is a regular internet user and has overall control with some minor problems), c) 50–79 for moderate internet use (one who has experienced occasional or frequent problems with internet use), and d) 80–100 points for severe internet use (one who has experienced significant problem in their life with internet use).

Both the IPAQ and IAT were translated into the local (Nepali) language with the help of a bilingual social scientist. Then, it was retroverted into the English language by an independent translator to ensure the original meaning of the scales remained the same. Pretesting of the tool was conducted among 10% of the estimated sample size. The pretested data was entered and analyzed, and necessary modification on the socio-demographic and technology-related portion of the questionnaire was done as required. The modification was done in consultation with the supervisor. However, the modifications were not made on standard tools i.e., IPAQ and IAT.

### Study variables

#### Dependent variables

The physical activity was categorized based on the level of intensity as adequate physical activity (IPAQ score ≥3000 MET-min/week) and inadequate physical activity (IPAQ score <3000 MET-min/week).

### Independent variables

Attributes were categorized as socio-demographic factors (age, gender, family type, father’s occupation, mother’s occupation, family income, pocket money), internet use (technological equipment access to house, access to the internet, online time, online activities, parental supervision) and educational factors (type of school, grade achieved in the last year, involvement in extracurricular activities).

### Data management and statistical analysis

Data were compiled, edited, and checked for consistency. EpiData (version 3.1) was used for data entry, and duplications and omissions were corrected before coding. Data were then exported to SPSS (version 21) for analysis. Descriptive statistics (frequency/ percentage) were calculated to study participant characteristics, and the Chi-square test was used to assess associations between dependent and independent variables. Bivariate logistic regression analysis was performed to find out the strength of association (unadjusted OR) between dependent and independent variables.

### Ethical considerations

Ethical approval for the study was obtained from the Institutional Review Committee of Pokhara University (Reference number 50/077/078). Additionally, permission was obtained from relevant authorities and educational institutions. Verbal and written consent was obtained from participants, ensuring confidentiality by using unique identification numbers instead of names. The collected information was exclusively used for the study.

## Results

### Socio-demographic and technology-related characteristics of the participants

‘Table 1” presents the socio-demographic and technology-related characteristics of the participants. Out of the 385 participants, more than half were in the 15–17 age group (54.3%), nearly half were male (52.5%), and the rest were female (47.5%). More than half of the participants belonged to nuclear families (55.8%). The father’s occupation was predominantly in agriculture (36.6%), followed by foreign labor (21.8%). Most of the respondents had a monthly family income between Rs. 40000 and Rs. 50000 (20.5%). The majority received monthly pocket money between Rs. 1000 and Rs. 5000 (56.9%). Twenty-one percent of students attended community schools. Participants largely achieved a B+ grade (31.7%), followed by A+ (25.2%) and A (24.7%). More than two-thirds of the participants (68.3%) were involved in extracurricular activities.

**Table 1:** Socio-demographic and technology-related characteristics of the participants (N = 385).

Variables	Number of Participants (N = 385)	Percentage (%)
**Age**		
15–17	209	54.3
17–19	176	45.7
**Sex**		
Male	202	52.5
Female	183	47.5
**Family type**		
Nuclear	215	55.8
Joint	140	36.4
Extended	30	7.8
**Father’s occupation**		
Agriculture	141	36.6
Foreign labor	84	21.8
Own business	58	15.1
Service: Government/Private	49	12.7
Foreign service	27	7
Daily wage laborer	25	6.5
Others	1	0.3
**Monthly family income (Rs.)**		
<10000	32	8.3
10000–19999	70	18.2
20000–29999	72	18.7
30000–39999	62	16.1
40000–49999	79	20.5
≥50000	70	18.2
**Monthly pocket money (Rs.)**		
<1000	83	21.6
1000–4999	219	56.9
5000–9999	80	20.8
10000–15000	3	0.8
**School type**		
Government School	153	39.7
Private school	150	39.0
Community School	82	21.3
**Grade achieved last year**		
A+	97	25.2
A	95	24.7
B+	122	31.7
B	49	12.7
C+	21	5.5
C	1	0.3
**Involvement in extracurricular activities**		
Yes	263	68.3
No	122	31.7
**Access to Internet**		
Yes	385	100.0
**Type of Internet**		
Wi-Fi	258	67.0
Cellular	127	33.0
**Device to use the internet**		
Mobile phones	374	97.1
Laptop and mobile	7	1.8
Laptop/Computer or tablets or smart TV	4	1.1
**Time spent on the Internet each day**		
<5 hour	252	65.5
≥5 hour	133	34.5
**Time spent on the internet for study each day**		
<2 hour	253	65.7
≥2 hour	132	34.3
**Time spent on the internet for social networking each day**		
<2 hour	334	86.8
≥2 hour	51	13.2
**Time spent on the internet for online gaming each day**		
<2 hour	337	87.5
≥2 hour	48	12.5
**Parental Control over the Internet**		
Yes	254	66.0
No	131	34.0

Among the 385 participants, almost all had access to internet service. Wi-Fi was the most used type of internet service (67%), and nearly all participants used a mobile phone for internet surfing (97.1%). About one-third of participants spent more than 5 hours online daily (34.5%). Almost two-thirds spent less than 2 hours a day on the internet for study purposes (65.7%), while the majority spent around 2 hours on social networking (86.8%). A minority spent more than 2 hours daily on online games (12.5%), and approximately two-thirds of participants had parental control over internet use (66%).

### Internet Addiction Test (IAT) score

“Table 2” depicts that most participants (70.4%) were mild internet users, while 15.6% were moderate internet users.

**Table 2:** Internet addiction test score (N = 385).

Internet addiction	Number of Participants (N = 385)	Percentage
(<20) No addiction	54	14
(20–49) Mild	271	70.4
(50–79) Moderate	60	15.6

### International Physical Activity Questionnaire (IPAQ) score

By calculating the total score for all participants’ activities, it was determined that almost three-fourths (75.3%) of the adolescents were inadequately physically active, as indicated in ‘Table 3”. Conversely, an IPAQ score of ≥3000 MET-minutes per week was considered indicative of being adequately physically active.

**Table 3:** International physical activity questionnaire score (N = 385).

IPAQ score	Number of Participants (N = 385)	Percentage
<3000 (Inadequate)	290	75.3
≥3000 (Adequate)	95	24.7

### Association between socio-demographic characteristics of participants and physical activity

"Table 4" displays the association between socio-demographic characteristics and physical activity. Pearson’s Chi-square test indicated a significant association between physical activity and the types of family (χ^2^ = 6.207, p-value = 0.045), occupation of the father (χ^2^ = 12.843, p-value = 0.046), monthly family income (χ^2^ = 30.412, p-value = <0.001), monthly pocket money (χ^2^ = 11.992, p-value = 0.007), and type of school (χ^2^ = 15.318, p-value = <0.001).

**Table 4:** Association between socio-demographic characteristics of participants and physical activity (N = 385).

Variable	Physical Activity (N = 385)		Total	Chi-square	df	p-value
	Inadequate n = 290 (75.3%)	Adequate n = 95 (24.7%)				
**Age group**						
15–17	154 (73.7%)	55 (26.3%)	209	0.662	1	0.416
17–19	136 (77.3%)	40 (22.7%)	176			
**Sex**						
Male	147 (72.8%)	55 (27.2%)	202	1.490	1	0.222
Female	143 (78.1%)	40 (21.9%)	183			
**Family type**						
Nuclear	109 (77.9%)	31 (22.1%)	140	6.207	2	0.045*
Joint	164 (76.3%)	51 (23.7%)	215			
Extended	17 (56.7%)	13 (43.3%)	30			
**Father’s occupation**						
Agriculture	96 (68.1%)	45 (31.9%)	141			
Service: Government/Private	39 (79.6%)	10 (20.4%)	49	12.843	5	0.046*
Own business	43 (74.1%)	15 (25.9%)	58			
Daily wage laborer	16 (64%)	9 (36%)	25			
Foreign labor	72 (85.7%)	12 (14.3%)	84			
Foreign service	23 (85.2%)	4 (14.8%)	27			
**Monthly family Income (Rs.)**						
<10000	16 (50%)	16 (50%)	32			
10000–19999	50 (71.4%)	20 (28.6%)	70			
20000–29999	46 (63.9%)	26 (36.1%)	72	30.412	5	<0.001*
30000–39999	50 (80.6%)	12 (19.4%)	62			
40000–49999	73 (92.4%)	6 (7.6%)	79			
≥50000	55 (78.6%)	15 (21.4%)	70			
**Monthly pocket money provided (Rs.)**						
<1000	51 (61.4%)	32 (38.6%)	83			
1000–4999	171 (78.1%)	48 (21.9%)	219	11.992	3	0.007*
5000–9999	65 (81.3%)	15 (18.8%)	80			
10000–15000	3	0	3			
**Type of school**						
Government	100 (65.4%)	53 (34.6%)	153			
Private	127 (84.7%)	23 (15.3%)	150	15.318	2	<0.001*
Community	63 (76.8%)	19 (23.3%)	82			
**Grade achieved last year**						
A+	77 (79.4%)	20 (20.6%)	97			
A	74 (77.9%)	21 (22.1%)	95	9.345	5	0.096
B+	94 (77%)	28 (23%)	122			
B	33 (67.3%)	16 (32.7%)	49			
C+	11 (52.4%)	10 (47.6%)	21			
C	1	0	1			
**Involvement in Extracurricular activities**						
Yes	202 (76.8%)	61 (23.2%)	263	0.980	1	0.322
No	88 (72.1%)	34 (27.9%)	122			

*Statistically significant at p<0.05

### Association of technology-related characteristics and Internet Addiction Test (IAT) score with physical activity

"Table 5" illustrates the association between technology-related characteristics, physical activity, and Internet Addiction Test (IAT) scores. Pearson’s Chi-square test revealed a significant association between physical activity and the type of internet access (χ^2^ = 5.902, p-value = 0.015) and Internet Addiction Test (IAT) score (χ^2^ = 6.721, p-value = 0.035).

**Table 5:** Association of technology-related characteristics and Internet Addiction Test (IAT) score with physical activity (N = 385).

Variable	Physical Activity		Total	Chi-square	df	p-value
	Inadequate 290 (75.3%)	Adequate 95 (24.7%)				
**Type of internet access**						
Wi-Fi	204 (79.1%)	54 (20.9%)	258	5.902	1	0.015*
Cellular	86 (67.7%)	41 (32.3%)	127			
**Time spent on the internet**						
<5 hour	194 (77%)	58 (23%)	252	1.081	1	0.299
≥5 hour	96 (72.2%)	37 (27.8%)	133			
**Time spent on the internet for study**						
<2 hour	195 (77.1%)	58 (22.9%)	253	1.216	1	0.270
≥2 hour	95 (72%)	37 (28%)	132			
**Time spent on the internet for social networking**						
<2 hour	249 (74.6%)	85 (25.4%)	334	0.812	1	0.367
≥2 hour	41 (80.4%)	10 (19.6%)	51			
**Time spent on the internet for online gaming**						
<2 hour	257 (76.3%)	80 (23.7%)	337	1.275	1	0.259
≥2 hour	33 (68.8%)	15 (31.3%)	48			
**Parental control over the internet**						
Yes	189 (74.4%)	65 (25.6%)	254	0.336	1	0.562
No	101 (77.1%)	30 (22.9%)	131			
**Internet Addiction Test (IAT) Score**						
<20	35 (64.8%)	19 (35.2%)	54			
20–49	214 (79%)	57 (21%)	271	6.721	2	0.035*
50–79	41 (68.3%)	19 (31.7%)	60			

*Statistically significant at p<0.05

### Strength of association

In the binary logistic regression (Table 6), it is evident that adolescents belonging to nuclear families (UOR = 2.689, 95% C.I = 1.178–6.136) and joint families (UOR = 2.459, 95% C.I = 1.119–5.405) were approximately two and a half times more likely to engage in inadequate physical activity compared to participants from extended families. Additionally, adolescents from families with a monthly income exceeding Rs. 50000 were over three times more likely (UOR = 3.318, 95% C.I = 1.209–9.105) to engage in inadequate physical activity compared to those with a monthly income less than Rs. 10000. Similarly, those using Wi-Fi were nearly twice as likely (UOR = 1.801, 95% C.I = 1.117–2.904) to engage in inadequate physical activity than adolescents using cellular internet service.

**Table 6:** Logistic regression analysis between physical activity and independent variables.

Variables	(OR)	95% CI	p-value
**Type of family**			
Nuclear	2.689	1.178–6.136	0.019*
Joint	2.459	1.119–5.405	0.025*
Extended	1		
**Monthly family income**			
<10000	1		
10000–19999	0.273	0.111–0.669	0.005*
20000–29999	0.682	0.315–1.474	0.330
30000–39999	0.483	0.229–1.018	0.056
40000–49999	1.136	0.486–2.659	0.768
≥50000	3.318	1.209–9.105	0.020*
**Type of school**			
Government	1		
Private	1.665	0.854–3.282	1.141
Community	0.569	0.309–1.049	0.071
**Type of internet access**			
Wi-Fi	1.801	1.117–2.904	0.016*
Cellular	1		
**Internet Addiction Test score**			
<20	1		
20–49	1.740	0.938–3.226	0.079
50–79	0.854	0.391–1.862	0.691

*Statistically significant at p<0.05

## Discussion

This study reveals that more than two-thirds of adolescents exhibited mild or moderate addiction to the internet. A comparable study among adolescents in selected schools in Pokhara, Nepal, showed that the majority of the total students included in the study were using the internet in their homes, and more than half of adolescents were using the internet for recreation purposes. Moreover, over half of these adolescents showed signs of moderate to severe internet addiction [30]. Internet use has grown exponentially worldwide, with the majority being adolescents and young people. In this study, the prevalence of inadequate physical activity among adolescents was quite high, consistent with the study conducted among more than a million adolescents by WHO, which shows that many students aged 11–17 years were insufficiently physically active. From the previous study, most adolescents do not meet current physical activity guidelines as recommended by WHO [4]. According to one of the studies conducted among adolescents from 105 low, middle, and high-income countries, the prevalence of adolescents doing sufficient physical activity was low, and very few (less than one-fifth) of the adolescents were engaged in physical activity every day, and the same portion (almost one-fifth) were never engaged in physical activity [31]. Also, a pooled analysis of 298 population-based surveys with 1.6 million participants revealed that a high portion of students were insufficiently physically active [4].

Moreover, this study states that adolescents whose age group is 17–19 were more likely to do inadequate physical activity than those whose age group is 15–17, the prevalence of inadequate physical activity among the 17–19 age group is higher than the 15–17 age group. Consistent with the study, a study conducted among adolescents in southern Brazil also stated that older students were likelier to do inadequate physical activity [32]. From these findings, it can be stated that middle adolescents are more likely to do adequate physical activity than late adolescents.

A significant association between family income and the physical activity level among adolescents was observed in this study. Adolescents from families with a monthly income of (≥50,000) were more likely to engage in inadequate physical activity compared to those from families with a monthly income of less than 10,000. In contrast, higher family income was associated with being an active sports club member and higher self-reported physical activity [33, 34]. A study from northern Finland stated that high family income was associated with adolescents becoming physically active [35]. Notably, problematic internet use rates tend to be higher among those with high household incomes, potentially due to increased access to personal devices, internet connections, and less involvement in physical activities [36]. High pocket money, indicative of high family income, often leads to increased internet use and reduced physical activity. On the contrary, adolescents from agricultural families, with limited internet access, were more likely to engage in adequate physical activity as they contributed to farming activities. Moreover, students in government schools, with limited computer and internet facilities, often utilize leisure time for games and sports and reported higher physical activity levels compared to those in private schools.

The type of internet service also demonstrated a significant association with physical activity. Adolescents using Wi-Fi were more likely to engage in inadequate physical activity than those using a cellular network. It can be assumed that the use of cellular networks provides more flexibility and is not restricted by Wi-Fi coverage, allowing for increased physical participation. This suggests that utilizing cellular networks for internet access may positively influence adolescents’ physical activity. However, no similar evidence was reported in previous studies to support this finding.

Additionally, this study found a significant association between Internet Addiction Test (IAT) scores and physical activity among adolescents. Smartphone addiction, as observed in a study among female university students, was negatively associated with physical activity i.e., students engaged in smartphone addiction were at higher risk of low physical activity [37]. The time spent on online learning also emerged as a significant factor explaining physical activity [38]. A study from Hong Kong revealed that internet gaming and smartphone addiction negatively impact physical activity, sleep, and academic performance [39]. Aligning with this study, a study among American adolescents highlighted that moderately active students who use the internet once or twice a month were more likely to report daily adequate exercise [40]. The evidence suggested that problematic internet use should be targeted as a means to improve physical activity [41]. In contrast to these findings, several works of literature also supported the beneficial effects of internet use on physical activity levels, though the determining factors warrant further investigation. It is evident that internet /smartphone use had a positive association with physical activity [42, 43]. So, the effect of internet use is not always detrimental.

The findings of this cross-sectional study are based on a minimal required sample size and a smaller setting. This study might not cover wider disciplines and factors and hence cannot be generalized for wider settings. So, future longitudinal studies with larger samples are necessary for further evidence on internet use and physical activity among Nepalese adolescents.

## Conclusions

In conclusion, this study found that over two-thirds of adolescents were addicted to the internet, and three-fourths were inadequately physically active, with a higher prevalence among middle adolescents compared to late adolescents. Inadequate physical activity was associated with family type, family income, occupation of the father, type of school, type of internet access, and Internet Addiction Test (IAT) score, respectively. Additionally, adolescents addicted to the internet were found to be physically inactive.

### Limitations

The language version of the tool used in the present study has not been validated.
